# Modeling the Effect of the Oxidation Status of the Ingredient Oil on Stability and Shelf Life of Low-Moisture Bakery Products: The Case Study of Crackers

**DOI:** 10.3390/foods9060749

**Published:** 2020-06-05

**Authors:** Lara Manzocco, Giulia Romano, Sonia Calligaris, Maria Cristina Nicoli

**Affiliations:** Department of Agri-Food, Animal and Environmental Sciences, University of Udine, via Sondrio 2/a, 33100 Udine, Italy; lara.manzocco@uniud.it (L.M.); giulia.romano@uniud.it (G.R.); mariacristina.nicoli@uniud.it (M.C.N.)

**Keywords:** Crackers, rancidity, lipid oxidation, shelf life, modeling

## Abstract

In packed low-moisture foods such as crackers, oxidation is generally the main cause of quality depletion during storage. It is commonly believed, but scarcely investigated, that product shelf life depends on the oxidative status of the lipid ingredients. In this study, the influence of oxidation degree of the ingredient sunflower oil on cracker oxidative stability and hence shelf life was investigated. To this aim, oil with increasing peroxide values (PVs) (5, 11, and 25 mEqO_2_/kg_oil_) was used to prepare crackers. Just after production, crackers presented similar peroxide and rancid odor intensity, probably due to the interactive pathways of oxidative and Maillard reactions. Crackers were packed and analyzed for PV and rancid odor during storage at 20, 40, and 60 °C. Rancid odor well discriminated cracker oxidative status. Relevant oxidation rates were used to develop a shelf life predictive model based on the peroxide value of the ingredient oil. It was estimated that an oil PV from 5 to 15 mEqO_2_/kg_oil_ shortens cracker Shelf Life (SL) by 50%, independently of storage temperature. These results demonstrate the critical impact of ingredient quality on product performance on the market.

## 1. Introduction

Primary shelf life is defined as a finite length of time after production and packaging during which food retains a required level of quality under specified storage conditions. As is well known, the term primary shelf life pertains exclusively to packed products, while the term secondary or pantry shelf life is conventionally used to indicate the lifetime of foods after pack opening [1,2]. In general terms, primary and secondary shelf lives depend on the rate at which food quality moves to reduced levels over time, until reaching the acceptability limit. The latter discriminates acceptable products from those no longer acceptable [3]. On its turn, the quality decay rate is the result of the interactive effects of different variables accounting for the intrinsic product characteristics (i.e., component concentrations, pH, water activity, presence of catalysts, etc.), packaging properties and performances, and storage conditions (temperature, light exposure, relative humidity, oxygen availability) [4,5,6].

Secondary shelf life and related issues have received little attention in literature even though their management could have important repercussions for consumers during home usage, but mostly for food managers facing the daily task of high-quality food production [7]. It is a matter of fact that the opening of packs or tanks after ingredient arrival at the warehouses determines a sudden cascade of changes, mainly due to the abrupt modification of the environmental conditions (e.g., loss of sterility, changes in oxygen concentration, moisture), leading to an acceleration of food quality decay rate and, in some cases, to the loss of product safety requirements. For these reasons, for a given product, the secondary shelf life is shorter than the primary one [7,8,9,10,11,12,13,14,15]. Although the quality of raw materials is routinely assessed at arrival at the industrial facilities, their quality depletion is rarely evaluated during industrial storage, even though their usage might be spread over a significant length of time after pack/tank opening. Keeping constant packaging and storage conditions, the quality level just after production of a formulated food mainly depends on the quality of the ingredients used to make it. Based on the same statement, it is reasonable that the quality status of ingredients can greatly affect the rate of quality decay of the product during storage and hence, its lifetime on the shelf.

Low-moisture bakery products, such as crackers, biscuits, and breadsticks, are generally characterized by long life on the shelves due to their low water activity. However, during storage, quality decay occurs due to a loss of crispness upon moisture adsorption or development of lipid oxidation [16]. Considering that the first phenomenon could be efficaciously faced by adopting proper packaging solutions, the control of lipid oxidation is more challenging. Oxidation is a complex set of reactions, initially involving lipids and oxygen, which further proceeds through the formation of a number of highly reactive radical species to finally result in undesirable off-flavors [17]. As is well known, also the addition of antioxidants in the formulation helps control lipid oxidation [18,19]. For these reasons, in shelf life assessment studies of low-moisture bakery products, lipid oxidation indexes are generally used [20,21,22,23]. Taking in mind the above reported considerations, the oxidation level of the lipid ingredients is expected to play a pivotal role in determining the shelf life of the finished products. Thus, as the oxidation level of the lipid ingredients increases, an acceleration of the oxidation rate of the bakery product on the shelf and a consequent reduction of product shelf life are expected. Although this statement sounds like common sense, to our knowledge, there is no literature evidence that demonstrates the link between these two aspects: quality of the ingredient vs. quality and shelf life of the final product. In other words, the assessment of primary and, when necessary, secondary shelf life of lipid ingredients represents a key factor to avoid overdating of the finished products and prevent undesired recalls. This would also turn producer efforts into a higher economic and environmental food sustainability since a higher food quality during storage is known to decrease the risk of food unacceptability and wasting by consumer [24,25].

The aim of this work was to assess the oxidative stability and relevant shelf life of packed crackers produced using sunflower oil having increasing oxidation levels. To this aim, sunflower oil samples showing different peroxide values were used to prepare three different cracker batches. The evolution of peroxide value and rancid odor intensity of cracker samples were monitored at 20, 40, and 60 °C and relevant oxidation rates were calculated. A model predicting cracker shelf life as a function of oil peroxide value was finally developed.

## 2. Materials and Methods

### 2.1. Raw Materials and Chemicals

Sunflower oil, type ‘0’ wheat flour, salt, and brewer’s yeast were purchased in local markets. Nonstabilized diethyl ether and 1-butanol were purchased by CARLO ERBA (Milan, Italy); 7-hydrate ferrous sulphate (FeSO_4_) by Merck KGaA (Darmstadt, Germany); anhydrous sodium sulphate (Na_2_SO_4_) and methanol by Sigma-Aldrich (Steinheim, Germany); 2,2,4-trimethylpentane (iso-octane) and hydrochloric acid by J.T. Baker (Deventer, Holland); 2-hydrate barium chloride (BaCl_2_) by EMSURE (Darmstadt, Germany).

### 2.2. Preparation of Sunflower Oil with Different Oxidation Levels

Aliquots of 500 mL of sunflower oil were inserted in 1 L capacity glass bottles in order to guarantee the presence of a sufficient oxygen concentration in the headspace. Samples were then stored at 60 °C in an incubator (Refrigerated Incubator FTC 90I for BOD, 230V, Velp Scientifica, Monza, Italy) for 0, 2, and 6 days in order to obtain sunflower oil batches with low, intermediate, and high oxidation levels, respectively. Oil samples were then immediately used for cracker formulation and baking.

### 2.3. Cracker preparation 

Cracker dough formulation was as follows: 500 g flour (61.43% *w*/*w* of the total amount), 180 g water (22.11% *w*/*w*), 120 g sunflower oil (14.74% *w*/*w*), 10 g salt (1.23% *w*/*w*), and 4 g brewer’s yeast (0.49% *w*/*w*). Sunflower oils with different oxidation levels prepared as described in paragraph 2.2 were used in the formulation. Three different cracker batches were prepared (low, intermediate, and high oxidation levels). Firstly, yeast was dispersed in half of the water, while salt was solubilized in the remaining part. Flour, oil having increasing oxidation level, yeast suspension, and salt solution were mixed in a dough mixer (Prospero KM260, 600 W, Kenwood, Havant, UK) for 2 min at medium speed and then for 3 min at the highest speed. The mixer provided optimum dough development by stretching it with a spiral kneading hook. The obtained dough was leavened for 2 h at 30 °C in an oven (Refrigerated Incubator FTC 90I for BOD, 230V, Velp Scientifica, Monza, Italy). Thereafter, the resulting cracker dough was sheeted using a dough-rolling machine (Titania, Imperia & Monferrina, Torino, Italy) to a thickness of 1.0 mm and cut into 4.0 cm × 6.0 cm rectangles. The latter were baked in an electric oven (AOS101ETA1, 17.5 KW, Electrolux Professional, Pordenone, Italy) for 17 min at 160 °C. After baking, crackers were allowed to cool at room temperature. Five individual crackers were packaged into 20 cm × 25 cm polylaminate aluminum/paper bags under atmospheric conditions using a laboratory sealer (VM-16, Orved, Venezia, Italy).

### 2.4. Cracker Storage

Fifteen packs of each batch of crackers (low, intermediate, and high oxidation levels) were stored in incubators at 20, 40, and 60 °C. At selected lengths of time during storage, one pack of each batch of samples with different oxidation levels were kept out from the three different incubators and analyzed for peroxide value and sensory rancidity level.

### 2.5. Analytical Determinations

#### 2.5.1. Moisture

Moisture content was determined by gravimetric method by drying the samples in a vacuum oven (1.32 kPa) at 75 °C until a constant weight was reached, according to the Gravimetric Official Methods of Analysis AOAC [26].

#### 2.5.2. Color

Color analyses were carried out on cracker images acquired by a digital camera (EOS 550D, Canon, Milan, Italy) using the program EOS UTILITY (version 3.30, Canon, Milan, Italy). The images were elaborated with the program Image Pro-Plus (version 6.3, Media Cybernetics, MD, USA). Color was expressed in L*, a*, b* Hunter parameters and a* and b* were used to compute hue angle [tan^−1^(b*/a*)] [27].

#### 2.5.3. Peroxide Value

Oil separation was carried out following the method of Kristensen et al. [28], with some modifications. Crackers were ground and mixed with iso-octane (1:3 *w*/*v*) by a high-speed homogenizer (Polytron PT-MR3000, Kinematica AG, Littau, Switzerland) for 4 min at 5000 rpm. Then, a mixture of purified diethyl ether, methanol, and iso-octane (2:1:1 *v*/*v*/v) was added to the homogenate in the ratio 1:6 (*w*/*v*). The dispersion was stirred at ambient temperature for 20 min (Yellow line MST basic, IKA-Werke GmbH & Co. KG, Staufen, Germany). The sample was filtered with filter paper (LABOR, Gruppo Cordenons, Milano, Italy) and dehydrated with sodium sulphate. Solvents were finally evaporated using a rotary evaporator (Rotavapor Laborota 4001, Heidolph, Schwabach, Germany) at 50 °C. The peroxide values (PVs) of the oil batches before use and the oil extracted from crackers were measured using the methods of Shantha and Decker [29] and Katsuda et al. [30]. In particular, oil was mixed with 2:1 (*v*/*v*) methanol/1-butanol solution in the ratio of 1:100 (*w*/*v*) and sonicated at 25 °C for 10 min (Ultrasonic Cleaner, VWR, Wayne, PA, USA). Then, 1.5 mL of sample was added with 8.4 mL of the methanol/1-butanol solution, 50 μL of ammonium thiocyanate solution (3.9 M), and 50 μL of 7-hydrate iron chloride solution (0.33 M barium chloride solution mixed with 0.036 M iron sulphate solution, dissolved 10 N hydrochloric acid). Samples were kept 20 min in the dark and then absorbance at 510 nm and 25 °C was analyzed (Shimadzu UV-250 1 PC, Kyoto, Japan). PV was calculated from the standard curve of 7-hydrate iron chloride in methanol/1-butanol solution. Relation between different iron concentrations and absorbance was studied and PV was calculated as follows:

PV(mEq O2kg)=(As−Ac)−ImWs∗55.84∗2

where As is sample absorbance, Ac is control absorbance, I is the intercept of standard curve, m is angular coefficient of standard curve, *Ws* is sample weight, and 55.84 is iron atomic weight.

#### 2.5.4. Rancid Odor

Aliquots of 5 g of crackers were placed into 75 mL capacity plastic containers, closed with a pressure cap, and labeled with a random three-digit code. Sensory analysis was performed according to Quantitative Descriptive Analysis^®^ technique. The samples were tested by a 30-member panel, approximately balanced between males and females with ages ranging from 20 to 50 years. The panelists were recruited among students and workers of the University of Udine, Italy. They were selected based on the criterion that they usually consumed crackers. Panelists were asked to sniff samples prepared by using fresh oil (control) and differently aged samples. Panelists judged the intensity of rancid odor of the unknown samples using a seven-point scale, anchored with four in correspondence of the control cracker. During each sensory session, the panelist tasted three unknown samples with 2 min resting time between them.

#### 2.5.5. Kinetics Data Analysis

Rate constants (*k*) relevant to peroxide value and rancid odor intensity formation as a function of storage time were calculated by applying a zero-order kinetic model [6]:

(1) $$PV=kt+PV_{0}$$

The effect of temperature on the rate of lipid oxidation was evaluated by means of the Arrhenius equation [31]. To make a better estimate of the apparent activation energy, a one-step nonlinear regression was applied to all data by using the reparametrized Arrhenius equation, in which was inserted a reference temperature chosen in the middle of the temperature range considered in the experimental plan:

(2) $$ln\;k=ln\;k_{ref}-\frac{E_{a}}{R}\left(\frac{1}{T}-\frac{1}{T_{ref}}\right)$$

where *k* is the apparent reaction rate, R is the molar gas constant (8.31 J/K/mol), *T* is the absolute temperature (K), and *k_ref_* is the apparent reaction rate at *T_ref_* (40 °C). *E_a_* and *k_ref_* were determined by linear regression analysis and used to calculate k_0_:

(3) $$k_{0}=e^{\left(\ln k_{ref}+\frac{E_{a}}{RT_{ref}}\right)}$$

#### 2.5.6. Shelf Life Computation

Cracker shelf life at a given temperature (*T*) was calculated using the following [6]:

(4) $$SL=\frac{\left(I_{lim}-I_{0}\right)}{k_{T}}$$

where *I*_0_ is the sensory rancid perception score of just-prepared crackers. *I_lim_* is the sensory rancid perception score corresponding to the acceptability limit (6 on a 7-point acceptability scale); *k_T_* is the sensory rancid perception rate constant.

### 2.6. Statistical Analysis

Data were expressed as the mean and standard deviation of at least two analytical determinations on two replicated samples. Statistical elaboration was performed with R Software (3.2.2 version, The R Foundation for Statistical Computing, Vienna, Austria). Bartlett’s test was used to check the homogeneity of variance, one-way ANOVA was carried out and Tukey’s HSD test was used as post hoc test to determine statistically significant differences among means (*p* < 0.05). Linear regression analysis was performed using Microsoft Excel 2016. The goodness of fitting was evaluated by using the coefficient of determination (R^2^), the standard error (SE), and the *p* value (*p*).

## 3. Results and Discussion

### 3.1. Effect of Oil Oxidation Level on Quality of Freshly Prepared Crackers

Three batches of sunflower oils having different initial peroxide values (5, 11, and 25 mEqO_2_/kg_oil_, respectively) were used to produce as many cracker samples. Such peroxide values were selected in order to simulate industrial situations in which the ingredient oil used for product preparation had increasing oxidation degree. For instance, a PV value of 11 mEqO_2_/kg_oil_ simulates the use of an oil with a quality status comparable to that recommended in the Official Codex Standards recommend as maximum acceptability limit for sunflower oil (10 mEqO_2_/kg_oil_, [32]). A PV of 25 mEqO_2_/kg_oil_, which largely exceeds the acceptability limit, was also chosen to stress as much as possible the effect of oil quality on cracker oxidation rate and shelf life. Table 1 shows moisture, colour, peroxide value (PV), and intensity of rancid odor of just-baked crackers prepared using oils with different oxidation degrees. 

No significant differences in terms of moisture and color were observed. In addition, independently of the oxidation degree of the oil used, freshly prepared crackers showed comparable peroxide value and rancid odor intensity. Surprisingly, these results do not seem to be reflecting the quality of the oil used as ingredient. The complex interplay of the phenomena taking place during dough preparation and baking might probably affect the evolution of primary oxidation products. In fact, the environmental conditions during processing (e.g., oxygen availability during dough leavening, baking temperature) are supposed to favor the oxidation of the lipid fraction and the consequent formation of hydroperoxides in the matrix. For this reason, when oil with a low oxidation degree is used, the final product is expected to show higher peroxide values than the oil [17,33,34]. By contrast, when the lipid ingredient is characterized by a very high oxidation level, a considerable number of primary oxidation products are accumulated in the matrix and their breakdown into secondary oxidation products, such as aldehydes, ketones, and other volatile compounds, represents the main reaction step during processing [35,36]. Moreover, during baking, lipid oxidation evolution could be influenced by the concomitant occurrence of the Maillard reaction involving sugars and proteins [37,38]. Different interactive pathways among these reactions have been reported in literature. On the one hand, Maillard reaction products, by virtue of their antioxidant capacity, can react as chain breakers and oxygen scavengers, slowing down the initial lipid oxidation step and thus hydroperoxide formation [38,39,40,41]. On the other hand, hydroperoxides were proven to enter Maillard reaction by forming secondary oxidation products, able to react with proteins [42,43,44].

### 3.2. Identification of the Quality Indicator for Cracker Shelf Life Assessment

**Figure 1:**
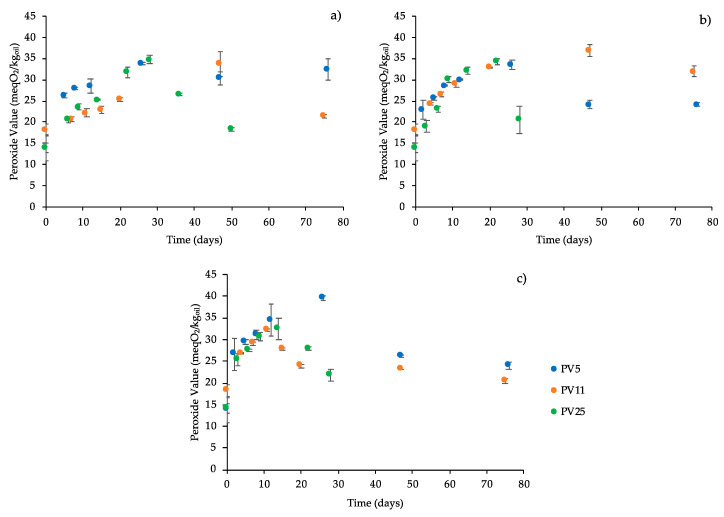
Peroxide value of crackers prepared with oil having different peroxide values (PVs, mEqO_2_/kg_oil_) as a function of storage time at 20 (**a**), 40 (**b**), and 60 °C (**c**). Error bars represent the standard deviation.

Based on the absence of a clear relation between oil oxidation status and potential quality indicators of cracker quality (Table 1), additional tests were carried out to select, between PV or rancid odor intensity, the best quality indicator to be used in a cracker shelf life study. To this aim, packed crackers were stored at ambient temperature (20 °C) to simulate the storage conditions on the market. Higher storage temperatures (40 and 60 °C) were also considered to force oxidative phenomena and emphasize the evolution of both quality indicators during storage. Figure 1 shows the PV evolution of cracker samples over storage time at the tested temperatures.

**Figure 2:**
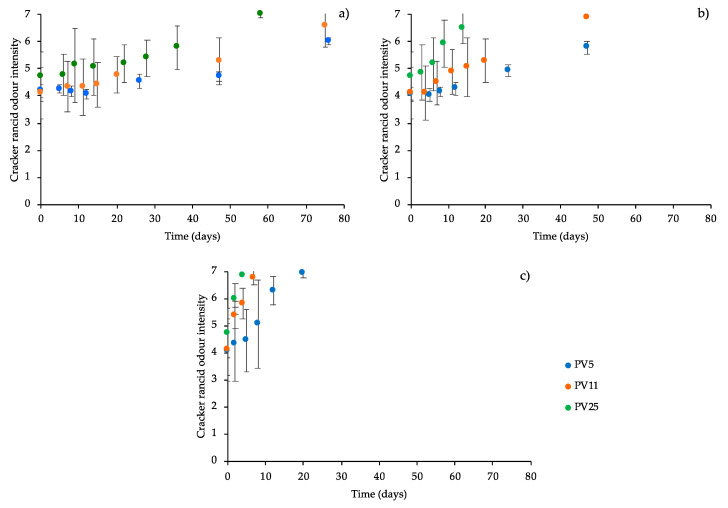
Sensory rancid odor perception relevant to crackers prepared with oil having different peroxide values (PVs, mEqO_2_/kg_oil_) as a function of storage time at 20 (**a**), 40 (**b**), and 60 °C (**c**). Error bars represent the standard deviation.

In all cases, a progressive PV increase, up to a maximum of around 40 meqO_2_/kg_oil_, was detected. However, a clear dependence between temperature and PV formation rate was not found and the temperature dependence of PV formation rate was not describable by applying the classic kinetic approach. Results indicate that PV evolution is not consistent with the oxidation degree of the oil used for cracker formulation. As previously mentioned, the absence of a clear relation between oil oxidation level and the evolution of oxidation in crackers during storage could be related to the complex and interactive reactions at the basis of hydroperoxides formation and decomposition [35,36,45,46,47]. Although the rise of PV indicates a progressive quality decay of the products during storage, this indicator was not suitable for discriminating the quality profile of the cracker batches both just after production and during storage. This result contradicts other literature data highlighting the goodness of PV as indicator to study oxidative status of bakery products [20,48]. It could be inferred that this behavior could be exactly attributed to the oxidation status of the ingredient oil. In other words, as the oil oxidation level increases, the reservoir of primary oxidation products in the matrix easily undergoes degradation, with formation of off-flavors. To verify this hypothesis, the changes of the intensity of rancid odor were monitored over time (Figure 2).

Results reported in Figure 2 actually show that the perception of sensory rancid odor progressively increased during storage for all samples. It is interesting to note that the perception of rancid odor resulted in being more sensitive than the monitoring of hexanal content in the headspace. In fact, preliminary results, performed by storing the samples at 60 °C, highlighted a significant increase of hexanal only after 20 days of storage (data not shown). It is evident observing Figure 2c that these samples were judged to have the maximum rancidity level. Rancid odor formation rate (*k*) was thus calculated by linear regression analysis according to the zero-order equation (Table 2).

It can be noted that cracker oxidation rates, expressed as rancid odor intensity, were consistent with the peroxide values of the oil used in the formulation; the higher the oil PV, the higher the rate of rancid odor formation at all storage temperatures. This result confirmed the hypothesis that as the initial PV reservoir increases, the development of rancid odor also increases.

**Figure 3:**
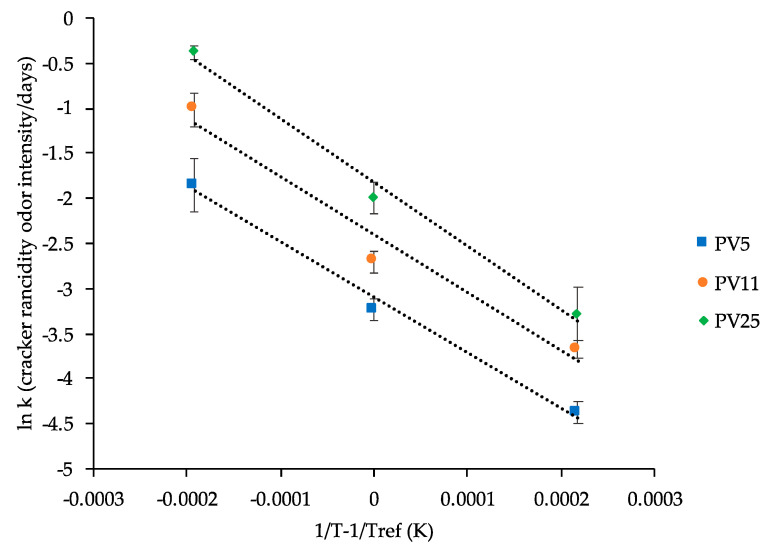
Temperature dependence of rates (*k*) relevant to sensory rancid perception in crackers prepared with oil having different peroxide values (PVs, mEqO_2_/kg_oil_). Error bars represent the standard deviation.

To further demonstrate the efficacy of this quality indicator in predicting the deterioration of the product during storage, the values of rancid odor formation rate (*k*) were plotted according to the Arrhenius model (Figure 3).

In all cases, the Arrhenius equation well described the temperature dependence of the cracker oxidation rate (R^2^ > 0.96). Activation energy (E_a_) and frequency factor (k_0_) were thus calculated by using Equations (1) and (2), respectively (Table 3).

Activation energy presented values consistent with data reported in literature for peroxide formation in sunflower oil [46,47,49]. Based on these sound results, the assessment of the sensory perception of rancid odor was selected as a proper quality indicator for the development of a shelf life model of crackers formulated with oil having different oxidation status.

### 3.3. Development of a Model Predicting Cracker Shelf Life as a Function of Oil Oxidation Status

Given the relevance of the sensory perception of rancid odor as indicator of cracker quality depletion, the rates of rancid odor formation at 20 °C were further analyzed to develop a model predicting cracker shelf life as a function of the peroxide value of the oil ingredient. Linear regression analysis demonstrated that the increase of rancid odor formation rate as a function of oil PV was well described (R^2^ = 0.99, *p* < 0.05) by the equation of a straight line:

(5) k=0.0012·PV+0.009

**Figure 4:**
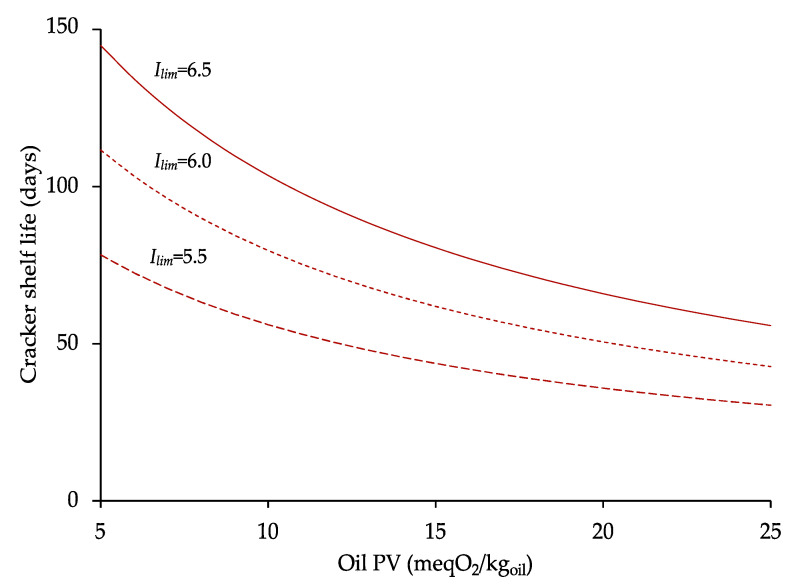
Estimated shelf life of crackers based on peroxide value of the ingredient oil and the rancid odor acceptability limit (I_lim_).

The obtained equation allowed the prediction of the rancid odor formation rate of crackers during storage on the basis of the peroxide values of the oil used in the formulation. Even if a validation considering other initial PV values would be required, this result strongly demonstrates the critical role of the oxidation level of the ingredient oil. For instance, the rate of odor formation increased by circa 30% for a 5 mEqO_2_/kg_oil_ enhancement of oil PV. This equation was combined with the well-known shelf life equation (Equation (3)) to develop a predictive tool estimating cracker shelf life as a function of oil PV.

(6) $$SL=\frac{I_{lim}-I_{0}}{k_{20\;{}^{\circ}C}}=\frac{I_{lim}-4.33}{0.0012\cdot PV+0.009}$$

where Io was taken as the average value of rancid odor intensity of just-baked crackers (Table 1). The model allows the cracker shelf life prediction to be obtained on the basis of two independent variables: (i) the peroxide value of the oil used in the formulation, within the PV range of 5 to 25 mEqO_2_/kg_oil_; (ii) the value of the acceptability limit, which is the rancid odor intensity chosen by the company to discriminate acceptable from no longer acceptable crackers. As reported in the literature, the choice of this value can have a dramatic impact on shelf life estimates [46]. Producers with high quality standards might tolerate only minimum changes in product rancid odor. By contrast, companies with a different market positioning could be prone to tolerating a higher rancid odor in the product at shelf life. Based on these considerations, Figure 4 visually shows the dependence of cracker shelf life (Equation (5)) as a function of the oxidation level of the ingredient oil and the acceptability limit selected by the producer. To this aim, acceptability limits corresponding to 5.5, 6.0, and 6.5 rancid odor scores were selected as examples of different company choices.

Results clearly demonstrate that company choices about the quality of both oil ingredient and final product dramatically affect the shelf life estimates. On the one hand, these figures can support the quick prediction of product shelf life based on company choices. On the other hand, they might be used to select the maximum PV of the oil ingredient, which is able to fit the production of crackers with the required quality level. For instance, should the target cracker shelf life be longer than 4 months with a tolerated rancid odor of 6 on a 7-point scale, the PV value of the oil ingredient should not be higher than 5 mEqO_2_/kg_oil_.

## 4. Conclusions

Based on the results acquired, the oxidative status of the lipid ingredients used in the industrial production of low-moisture bakery products should be carefully monitored not only upon arrival of the ingredient but also during its subsequent storage within the company. In the case of crackers containing sunflower oil, rancid odor was found to be the best indicator able to discriminate cracker oxidative status and relevant oxidation rates. This indicator can be used to develop a shelf life predictive model based on the peroxide value of the ingredient oil. It was estimated that an oil PV from 5 to 15 mEqO_2_/kg_oil_ shortens cracker shelf life by 50%, independently of storage temperature. Even if more research is needed to validate the model and adapt the proposed procedure to other low-moisture products, results highlighted the key role of the oxidative status of lipid ingredients in assuring a constant quality of the products just after production and a reliable dating. Such consideration is particularly true when the use of lipid ingredients is spread over a significant length of time after pack opening.

## Figures and Tables

**Table 1:** Moisture, color, peroxide value (PV), and intensity of rancid odor of just-prepared crackers containing oil having increasing oxidation level.

Oil	Cracker				
	Oil PV (mEqO_2_/kg_oil_)	Moisture (% p/p)	Hue Angle (tg^−1^ (b*/a*))	PV (mEqO_2_/kg_oil_)	Intensity of Rancid Odor
Low	5.06 ± 0.21	2.40 ± 0.63 ^a^	107.00 ± 1.29 ^a^	13.86 ± 3.06 ^b^	4.17 ± 1.08 ^a^
Intermediate	11.34 ± 0.63	2.45 ± 0.08 ^a^	108.72 ± 1.06 ^a^	18.18 ± 1.48 ^a^	4.10 ± 0.95 ^a^
High	25.22 ± 0.48	2.39 ± 0.13 ^a^	106.78 ± 1.63 ^a^	14.02 ± 1.10 ^b^	4.72 ± 0.90 ^a^

a,b: For each parameter, different letters show significant differences (*p* > 0.05).

**Table 2:** Zero-order rate constants of rancid odor formation (*k*) and corresponding regression parameters of crackers prepared with oil having different peroxide values (PV, mEqO_2_/kg_oil_) and stored at increasing temperatures.

Oil PV (mEqO_2_/kg_oil_)	Temperature (°C)	*k* (Rancid Odor Intensity/Day)	Standard Error	R^2^
5	20	0.013	0.003	0.81
	40	0.040	0.003	0.98
	60	0.156	0.018	0.95
11	20	0.025	0.003	0.95
	40	0.067	0.003	0.99
	60	0.363	0.017	0.95
25	20	0.038	0.018	0.95
	40	0.136	0.017	0.95
	60	0.684	0.033	0.99

**Table 3:** Frequency factor (*k_o_*), activation energy (*Ea*), and corresponding regression parameters of rancid odor formation in crackers produced with oil having different peroxide values (PVs).

Oil PV (mEqO_2_/kg_oil_)	*k_o_* (Rancid Odor Intensity/Day)	*E_a_* (kJ/mol)	R^2^	*p*
5	1.37·10^7^	50.82	0.99	0.06
11	7.81·10^7^	53.53	0.96	0.12
25	2.48·10^8^	58.53	0.99	0.06
